# In vivo activation of latent HIV with a synthetic bryostatin analog effects both latent cell "kick" and "kill" in strategy for virus eradication

**DOI:** 10.1371/journal.ppat.1006575

**Published:** 2017-09-21

**Authors:** Matthew D. Marsden, Brian A. Loy, Xiaomeng Wu, Christina M. Ramirez, Adam J. Schrier, Danielle Murray, Akira Shimizu, Steven M. Ryckbosch, Katherine E. Near, Tae-Wook Chun, Paul A. Wender, Jerome A. Zack

**Affiliations:** 1 Department of Medicine, Division of Hematology and Oncology, University of California Los Angeles, Los Angeles, California, United States of America; 2 Department of Chemistry and Department of Chemical and Systems Biology, Stanford University, Stanford, California, United States of America; 3 Department of Microbiology, Immunology, and Molecular Genetics, University of California Los Angeles, Los Angeles, California, United States of America; 4 Department of Biostatistics, School of Public Health, University of California Los Angeles, Los Angeles, California, United States of America; 5 National Institute of Allergy and Infectious Diseases, National Institutes of Health, Bethesda, Maryland, United States of America; University of North Carolina at Chapel Hill, UNITED STATES

## Abstract

The ability of HIV to establish a long-lived latent infection within resting CD4+ T cells leads to persistence and episodic resupply of the virus in patients treated with antiretroviral therapy (ART), thereby preventing eradication of the disease. Protein kinase C (PKC) modulators such as bryostatin 1 can activate these latently infected cells, potentially leading to their elimination by virus-mediated cytopathic effects, the host’s immune response and/or therapeutic strategies targeting cells actively expressing virus. While research in this area has focused heavily on naturally-occurring PKC modulators, their study has been hampered by their limited and variable availability, and equally significantly by sub-optimal activity and *in vivo* tolerability. Here we show that a designed, synthetically-accessible analog of bryostatin 1 is better-tolerated *in vivo* when compared with the naturally-occurring product and potently induces HIV expression from latency in humanized BLT mice, a proven and important model for studying HIV persistence and pathogenesis *in vivo*. Importantly, this induction of virus expression causes some of the newly HIV-expressing cells to die. Thus, designed, synthetically-accessible, tunable, and efficacious bryostatin analogs can mediate both a “kick” and “kill” response in latently-infected cells and exhibit improved tolerability, therefore showing unique promise as clinical adjuvants for HIV eradication.

## Author summary

HIV can persist for many years in a latent (non-expressing) state in individuals treated with antiretroviral therapy, and these latently-infected cells represent a major barrier to curing HIV infection. One potential approach to eliminating the reservoir cells is to induce them to express viral proteins while maintaining antiretroviral therapy, allowing these cells to be killed by the virus or the immune response. However, this approach has been hampered by a lack of compounds that can safely and effectively reverse HIV from latency in a living organism. Here we show that a designed, synthetic protein kinase C modulating compound is better-tolerated than the naturally-occurring product (bryostatin 1) in mice and potently induces HIV expression from latency in a mouse model containing a human immune system. Importantly, this induction of virus expression causes some of the newly HIV-expressing cells to die. Hence, this compound shows unique promise as a component of the so-called “kick and kill” approach for HIV eradication.

## Introduction

HIV/AIDS is a catastrophic pandemic that has claimed an estimated 35 million lives. Approximately 37 million individuals are currently HIV-positive. In 2015, 2.1 million individuals were newly infected, and 1.1 million died of AIDS-related illnesses [1]. Antiretroviral therapy (ART), the current recommended treatment for HIV infection, suppresses viral replication and prevents disease progression, allowing infected individuals to live with the disease [2]. However, ART does not cure the infection, in part because replication-competent HIV can persist within latently-infected CD4+ T cells throughout many years of continuous therapy [3,4,5]. These reservoir cells contain integrated HIV proviral genomes and can episodically re-supply active virus. Treatment thus requires the life-long use of ART, which is associated with numerous problems including health issues related to chronic chemoexposure, high financial cost and the need for strict compliance [6]. The development of strategies to reduce or eliminate the reservoir of latently-infected cells is therefore a research and clinical priority of global significance.

Several strategies for eliminating latent HIV have been proposed (reviewed in [7]). One promising approach is often referred to as “activation-elimination” or “kick and kill”. This is based on the observation that latently-infected cells do not typically express viral proteins and are therefore not killed directly by virus production (viral cytopathic effects) or through immune effector mechanisms such as cytotoxic T lymphocytes (CTL) or natural killer cells, which require viral protein expression to recognize infected cells. However, if HIV expression can be induced in latently-infected cells (kick) then these cells could become susceptible to cytopathic effects or virus- or immune-mediated cell killing mechanisms (kill). It is currently unknown whether inducing expression of latent HIV *in vivo* alone is sufficient to deplete some or all latently-infected cells, or whether the “kill” arm of the approach will require augmenting, for example, with broadly-neutralizing anti-HIV antibodies [8,9], anti-HIV immunotoxins [10], pre-stimulated or genetically engineered CTLs [11,12], or other mechanisms. The capacity of a particular stimulatory signal to result in the death of latently-infected cells is likely connected to its relative ability to induce HIV protein expression, with weak HIV latency reversing agents (LRAs) inducing little or no protein expression, and strong LRAs potentially inducing expression of sufficient HIV protein to trigger viral cytopathicity and/or immune surveillance in the host. However, this approach is further complicated because HIV expression is tightly connected to the activation state of the host CD4+ T cell, meaning that very strong LRAs might also induce CD4+ T cell activation, proliferation, and/or generalized immune stimulation accompanied by hypercytokinemia (cytokine storm) such as can occur following *in vivo* administration of the anti-CD3 antibody OKT3 along with interleukin (IL)-2 [13]. Therefore, an ideal LRA would strongly induce HIV expression *and* cause the death of latently-infected cells without over-activating immune cells.

Numerous studies on agents that induce latency reversal through several different cellular pathways have been reported [2,14,15,16]. Of these, protein kinase C (PKC) modulators are an especially promising LRA class of preclinical leads, serving either as single agents or in combination with additional LRAs [2,14,17,18]. The vast majority of prior *in vitro* HIV studies with PKC modulators has focused on one of the first reported LRAs, naturally-occurring prostratin, with a more recent interest being directed also at ingenol esters and bryostatin 1 [19,20].

**Fig 1:**
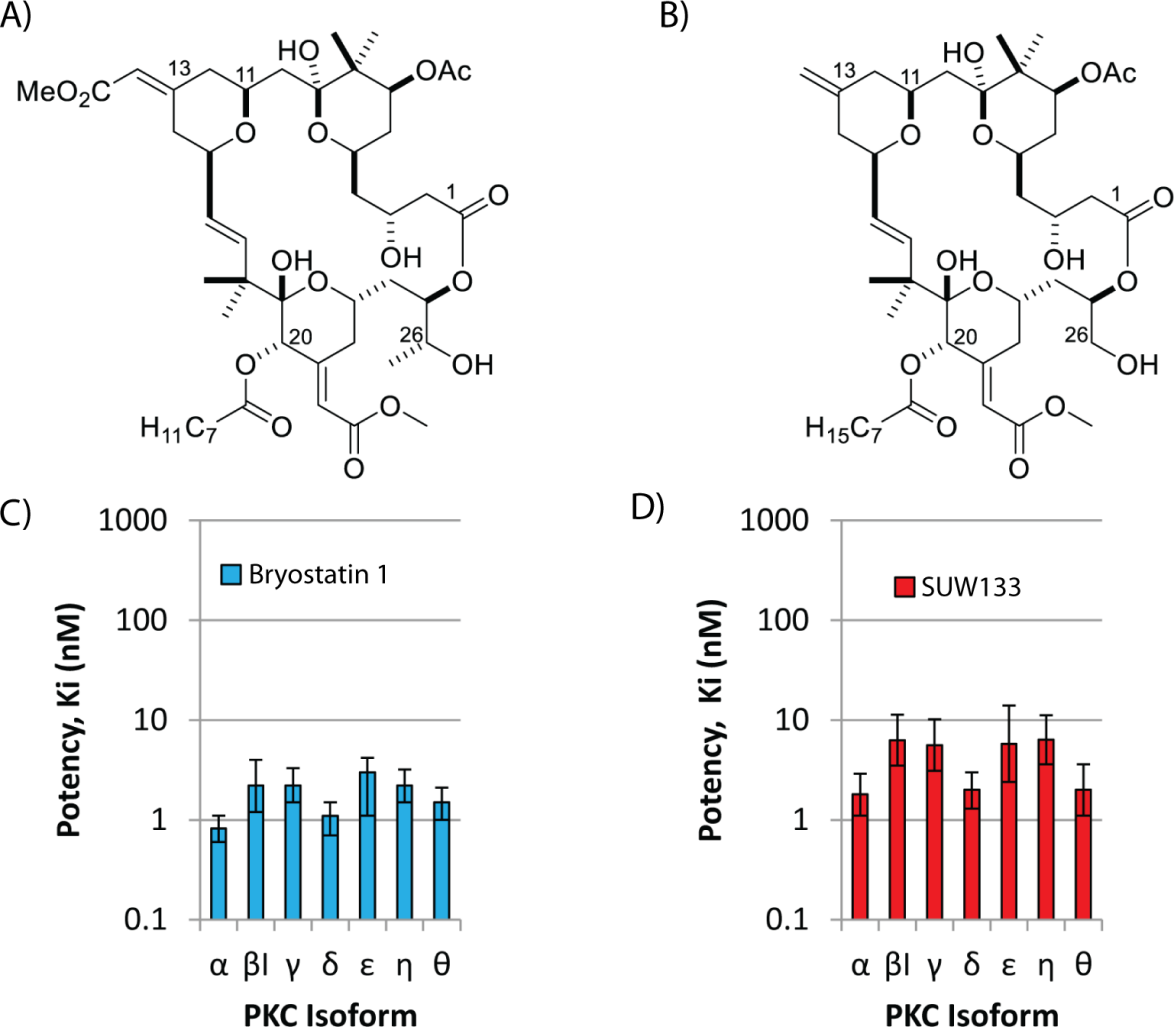
A synthetic bryostatin analog exhibits pan-PKC binding selectivity similar to bryostatin 1. **A)** Structure of bryostatin 1. **B)** Structure of bryostatin analog SUW133. **C) & D)** Affinities of bryostatin 1 and SUW133 in binding to conventional and novel PKC isoforms.

The bryostatins are a collection of at least 21 structurally related macrolactone natural products originally isolated from the marine bryozoan *Bugula neritina* [21]. Bryostatin 1 (Fig 1A), the most studied of the naturally occurring bryostatins [22], modulates PKC activity at low nanomolar concentrations and is implicated in a broad range of biological activities. Its use in cancer therapy has been explored in over 40 phase I and phase II clinical trials. It has also been studied in a phase IIb trial [23] for moderate-to-severe Alzheimer’s disease [24,25]. Additionally, bryostatin 1 potently induces HIV from latency in various *in vitro* models [17,26], and is therefore a lead clinical candidate in HIV eradication efforts. This positive *in vitro* activity prompted a recent phase I clinical study in ART-treated patients, which showed that bryostatin 1 was safe at low doses, but higher doses would be required to effect PKC-mediated latency reversal [27]. The potential of lowering the dose of bryostatin 1 and thus increasing its tolerability with combination LRAs was not explored.

Notwithstanding its clinical potential, the supply of bryostatin 1 is uncertain as it is produced in only low and variable amounts by its marine source organism. Sustainable harvesting of that source raises cost and environmental concerns. Most importantly, the natural bryostatins, serving putatively in part as antifeedants in their marine ecosystem, are neither evolved, optimized nor readily tuned for therapeutic applications such as HIV latency reversal. Indeed, natural products themselves represent only a small percentage (6%) of new chemical entities introduced as drugs with the vast majority being derivatives or agents inspired by natural products [28].

To address in part the limited availability of natural products, the difficulty often encountered in their chemical derivatization due to their structural complexity and scarcity, and their generally unoptimized clinical potential, we have focused on a function oriented synthesis (FOS) strategy directed at creating therapeutic function through synthesis-informed design [29]. In brief, rather than focusing on structure alone, which is an all-or-nothing approach, FOS focuses on function which could be achieved with a wide range of structures through innovative design. Toward this end, based on a computer analysis of PKC modulators, we previously proposed [30] that only a subset of features in the complex bryostatin 1 structure might be responsible for its biological activity, and used [26] this pharmacophore model to design the first simplified and more synthetically accessible bryostatin analogs (“bryologs”). Here, we report on the ability of novel bryologs to function like bryostatin 1 in activating viral gene expression from the actual latent HIV reservoir and to quantify *in vivo* activity and tolerability of one particularly promising analog (SUW133) as required for its preclinical advancement. In a clear demonstration of the potential value of the FOS approach, we show that this bryolog is somewhat better tolerated *in vivo* than bryostatin 1, that it is more effective in activating expression of latent HIV in human cells, and, significantly, that this expression (“kick”) causes a subset of these infected host cells to die without requiring an additional "kill" approach.

## Results

**Fig 2:**
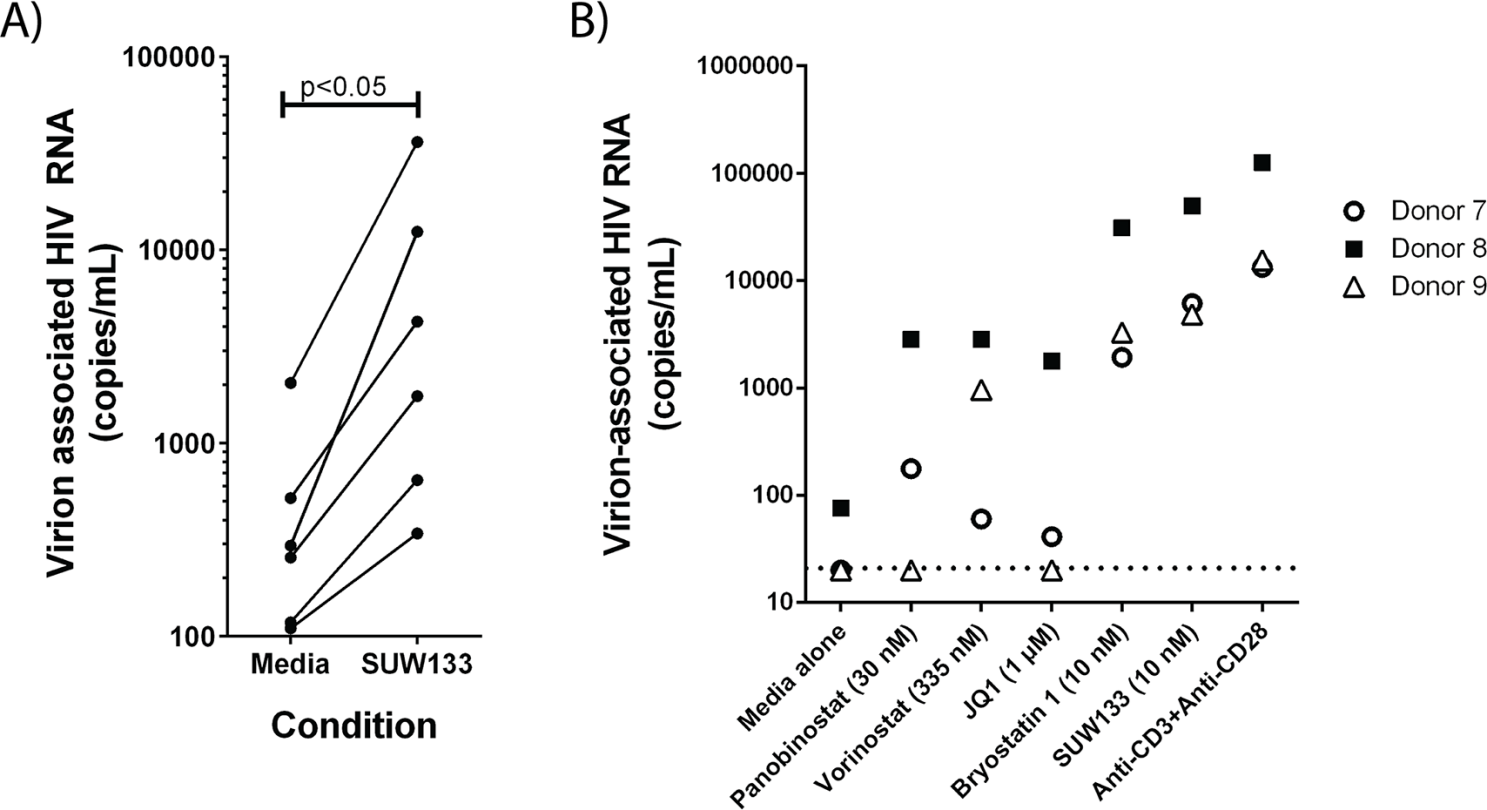
Activation of HIV from latency in patient-derived cells. CD4+ T cells from ART-treated patients (described in S1 Table) were isolated and exposed to SUW133 or other stimuli before quantification of cell-free virion-associated viral RNA levels in the culture supernatant. **A)** Resting CD4+ T cells from 6 aviremic patients were stimulated for 36h with media alone or 50 nM of SUW133, p value was calculated using a 2-sided Wilcoxon rank sum test. **B)** CD4+ T cells from an additional 3 ART-treated, aviremic patients were subjected to stimulation for 48h with the indicated compounds. Using a rank sum test (for independence), SUW133 induced significantly higher (p = 0.03) amounts of virus production versus all other stimuli except maximal T cell stimulation (anti-CD3 + anti-CD28 costimulation).

The structures of bryostatin 1 and bryolog SUW133 along with PKC isoform affinities are shown (Fig 1). In a preliminary screen for LRA activity, we previously demonstrated that SUW133 and several other bryologs effectively induced HIV from latency in the J-Lat 10.6 cell line [26]. Since HIV latency reversal varies depending on the model system used, and evaluating potential LRAs using primary latently-infected cells from ART-treated patients is an important step in their characterization, we have now tested SUW133 for its capacity to induce virus expression from resting CD4+ T cells obtained from six HIV-infected patients with viral loads suppressed by effective ART (Subjects 1–6, S1 Table). SUW133 induced significant levels of latent virus from these true reservoir cells (Fig 2A) as well as in an alternative cell line model for HIV latency (U1 cells: S1 Fig). To directly compare SUW133 treatment with other commonly tested HIV LRAs and maximal T cell stimulation, CD4+ T cells were isolated from a further 3 ART-treated patients with undetectable plasma viral loads (Subjects 7–9, S1 Table). These cells were exposed to clinically relevant concentrations [31] of the histone deacetylase inhibitors panobinostat or vorinostat, the BET bromodomain inhibitor JQ1, the PKC modulator bryostatin 1, or anti-CD3+anti-CD28 antibody costimulation (Fig 2B). SUW133 significantly outperformed panobinostat, vorinostat, JQ1, and bryostatin 1 treatment in this assay, and induced approximately 1/3 the amount of cell-free virion-associated HIV RNA as maximal T cell stimulation (costimulation). These data indicate that SUW133 is capable of inducing HIV from latency in primary patient-derived latently-infected cells.

**Fig 3:**
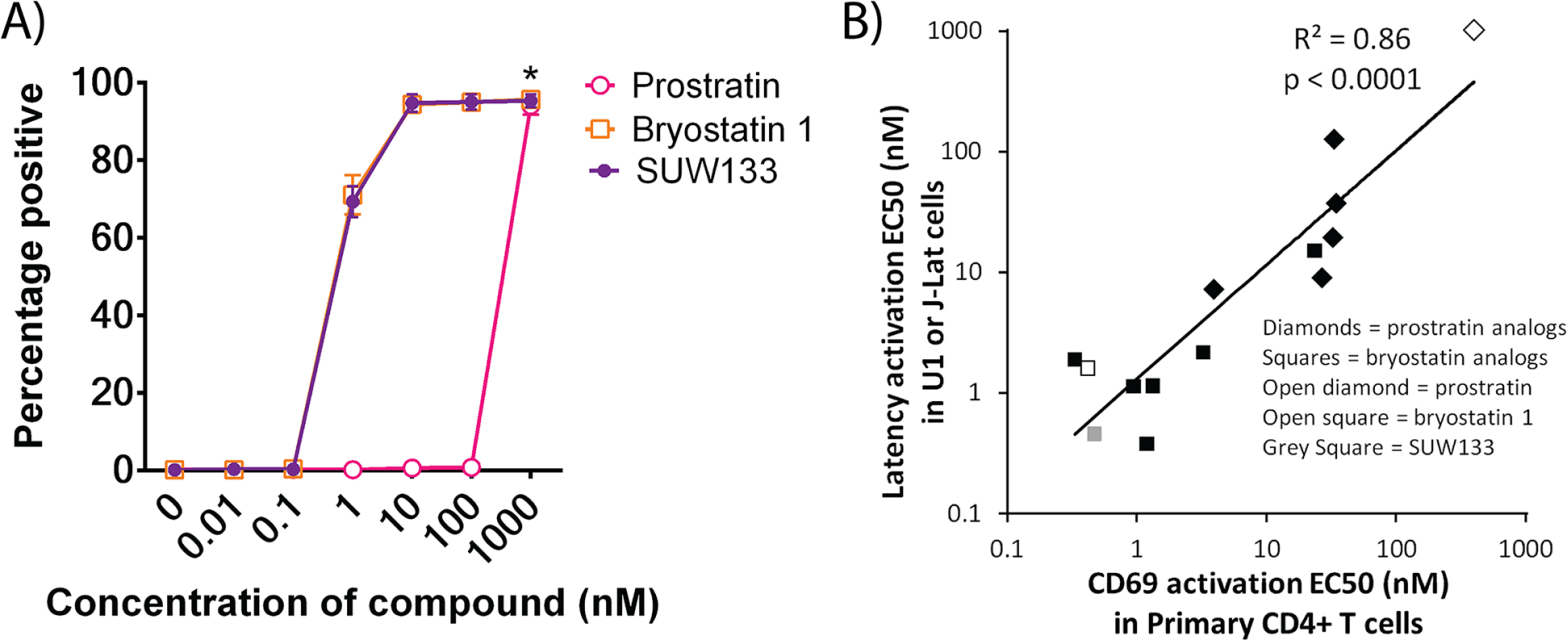
Induction of CD69 expression by compounds in primary CD4+T cells. **A)** Isolated CD4+ T cells from healthy donors were stimulated for 24h with the indicated compound and then analyzed for CD69 expression by flow cytometry. Error bars represent ±1 Standard Error (N = 3 different primary cell donors). * Indicates p = 0.05 for all tested compounds in untreated vs. 1000 nM treatment conditions (1-sided Wilcoxon rank sum test). **B)** EC_50_ comparison showing correlation between HIV latency activation and induction of CD69 expression by PKC activators. Data for prostratin analogs are from [18], and data for bryostatin analogs are from [26] and this study. Data were analyzed by linear regression. The p-value reflects evidence against the null hypothesis of no effect (i.e. that the regression coefficient is zero). The p-value being below 0.05 suggests that CD69 activation is significantly related to latency activation.

Prostratin and bryostatin 1 can each induce expression of the early T cell activation marker CD69. We found that SUW133 also upregulates expression of this cell surface marker on primary CD4+ T cells (Fig 3A), and that for a variety of synthetic prostratin and bryostatin analogs [18,26] this effect occurs at similar concentrations to those required to activate HIV from latency (Fig 3B). This indicates that CD69 serves as an *in vivo* biomarker to assess when biologically active concentrations of a compound have been delivered and thus can be used to evaluate the compound’s relative potency.

**Fig 4:**
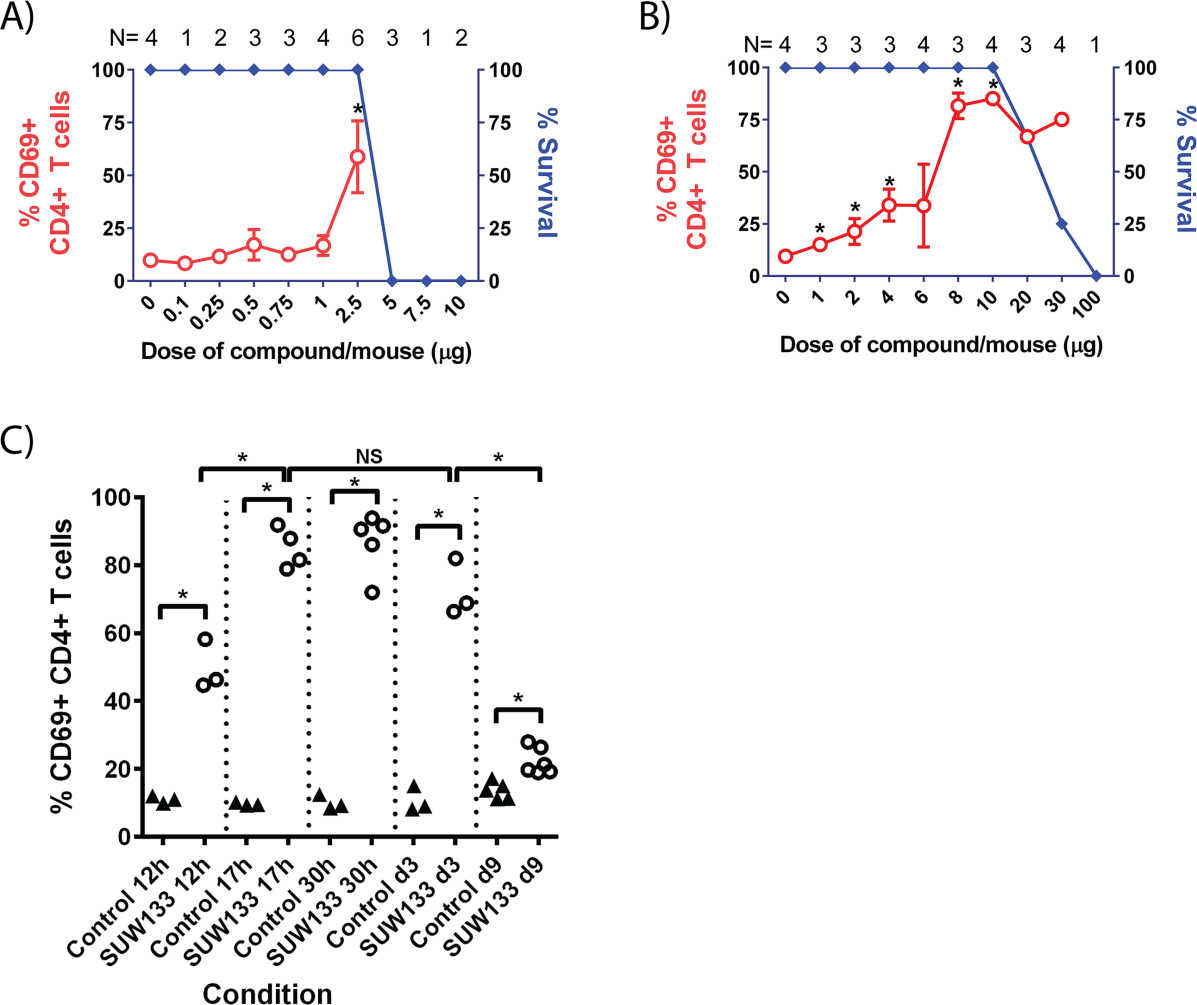
*In vivo* stimulation in C57bl6 mice. **A)** *In vivo* toxicity and bioactivity (CD69 upregulation in splenocytes) of bryostatin 1 at different doses of compound in C57/bl6 mice. The “% survival” indicates the percent of mice that survived the 17-24h overnight time course. Error Bars = SEM, *p = 0.05 (One-sided Wilcoxon rank sum test of treatment versus control [0 dose]). The number of mice (N) used for each comparison is given at the top of the figure. **B)** Comparative bioactivity and tolerability of bryolog SUW133 in C57/bl6 mice. Statistics and labels are as described in part A. **C)** Time course (12h, 24h, 30h, 3 days, and 9 days post-stimulation) of CD69 activation in splenocytes following *in vivo* administration of SUW133. Each data-point represents a different mouse (3–6 mice per group. *p = 0.05 (One-sided Wilcoxon rank sum test of treatment versus control).

To determine whether the PKC modulator SUW133 has improved properties over bryostatin 1 *in vivo*, ascending concentrations of bryostatin 1 or SUW133 were administered by intraperitoneal injection into C57/bl6 mice and the relative *in vivo* bioactivity and acute toxicity of the compounds were evaluated. Both bryostatin 1 and SUW133 induced high levels of CD69 in murine splenocytes at 24h post-injection. However, SUW133 exhibited superior activity, with over 80% of CD4+ splenocytes expressing CD69 versus a maximum of approximately 60% in bryostatin 1 treated animals (Fig 4A and 4B). Importantly, while bryostatin 1 had a very narrow toxicity free activation window between 2.5 μg and 5 μg per animal, SUW133 induced CD69 expression at doses as low as 1 μg and in some cases was tolerated at doses of up to 30 μg per animal. Thus SUW133 exhibits a moderately greater “therapeutic window” than the natural product and better induction, showing that toxicity and activation can be decoupled by structural variations.

HIV latency purging strategies would ideally involve a transient “pulse” of stimulation that would be sufficient to induce activation and elimination of a portion of latent virus reservoir cells without causing persistent immune activation. Repetition of this treatment could eventually eliminate the latent reservoir. A single injection of 10 μg of SUW133 indeed resulted in transient expression of CD69 on the vast majority of murine splenocytes (Fig 4C) which declined by day 9 post-injection. CD25 (a late T cell activation marker and component of the IL-2 receptor) expression on CD3+ T cells in the spleens of these animals remained unchanged (S2 Fig). Moreover, no detectable increase in plasma concentrations of TNF-alpha, interleukin (IL)-2, and MIP-1-alpha, and only a short-term elevation in IL-6 levels was observed following administration (S3 Fig).

**Fig 5:**
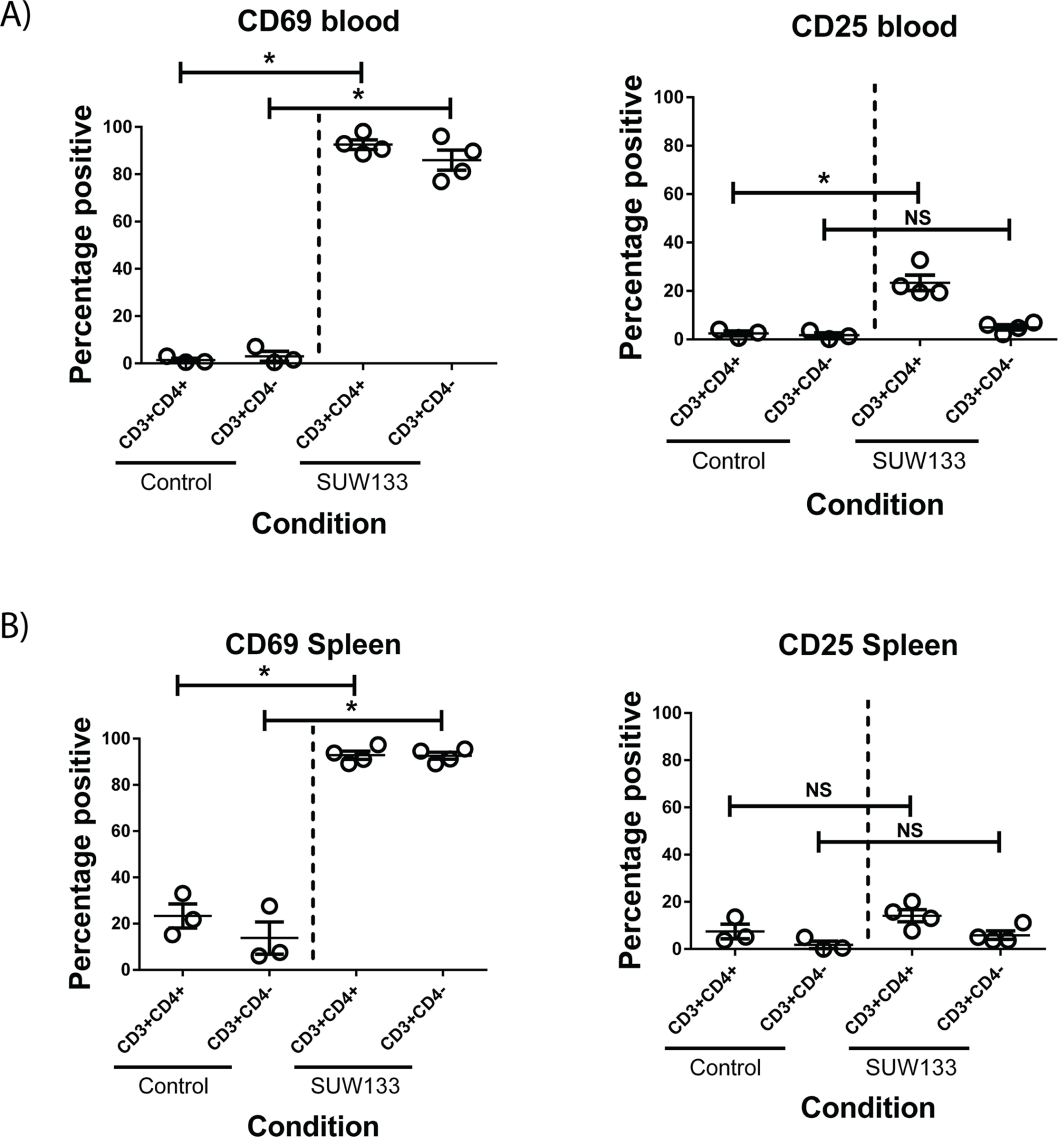
Bryolog induces stimulation of human cells *in vivo*. Humanized BLT mice were injected with SUW133 and the blood and spleen evaluated 24h later for human cells expressing activation markers. **A)** % CD69+ cells or CD25+ cells in different human T cell subsets in peripheral blood. **B)** % CD69+ cells or %CD25+ cells in different human T cell subsets in spleen. N = 3 mice/group control and 4 mice/group SUW133. *p<0.05 (Wilcoxon rank sum test).

We next explored the activity of SUW133 in humanized mice, a proven, important and relevant model for studying HIV *in vivo*. The BLT mouse model [32] supports multi-lineage human immune reconstitution in many tissues within the mouse and represents one of the most advanced small animal models available for investigating HIV persistence and pathogenesis [33]. We and others have shown that BLT mice can be infected with HIV and form authentic post-integration latency in resting human CD4+ T cells [34,35]. We therefore elected to use this model to determine whether SUW133 could induce latent HIV expression *in vivo*. We initially verified the bioactivity of SUW133 in human cells in uninfected BLT mice by quantifying activation of CD69 expression in the spleen and blood one day after stimulation (Fig 5). This resulted in robust activation of CD69 with limited CD25 expression in both CD4+ T cells and other T cell subsets. Given the strong correlation between HIV latency reversal and CD69 upregulation we had previously identified (Fig 3), these data suggested that SUW133 would also be capable of inducing expression of latent HIV *in vivo*.

**Fig 6:**
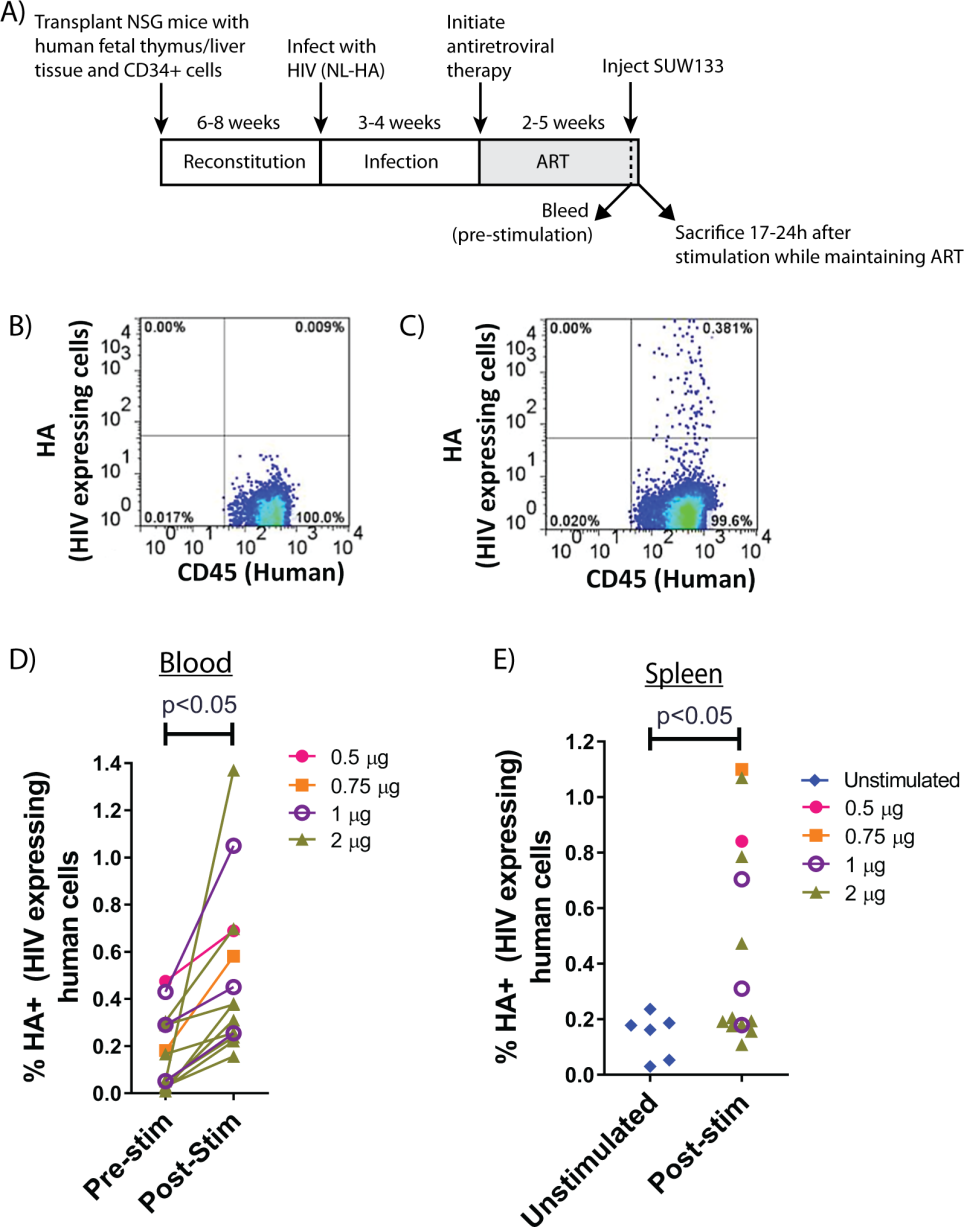
Synthetic bryolog can induce expression of latent HIV *in vivo* in humanized mice. **A)** Experimental scheme: Humanized BLT mice were infected with HIV strain NL-HA, which expresses the HA epitope on the surface of productively-infected cells (including those induced to express from latency). Infected mice were treated with ART (emtricitibine, tenofovir disoproxil fumarate, and raltegravir) for 3–5 weeks to suppress viral loads. Mice were then bled to obtain a baseline data point for HA expression and treated with a single dose of SUW133. After an overnight incubation, mice were sacrificed and the frequency of HIV-expressing human cells re-assessed in blood and spleen. **B)** Example flow cytometry plot showing low numbers of HA+ (HIV-expressing) human cells in the peripheral blood of ART-suppressed mice. **C)** Peripheral blood staining (from the same animal show in in panel B) 17h after administration of SUW133, showing increase in HA+ (HIV-expressing) human cells. **D)** Percentage of HA+ human cells in the peripheral blood before and after administration of SUW133 (each line represents an individual mouse). **E)** Percentage of human HA+ (HIV-expressing) cells in spleen of unstimulated ART-treated infected animals or those that received the indicated dose of SUW133 (each data-point represents an individual mouse). P-values were determined using a 2-sided Wilcoxon sign rank test for paired data.

To test this hypothesis, we then infected BLT mice with NL-HA, a replication-competent, pathogenic HIV reporter strain that encodes a short epitope from influenza hemagglutinin (HA) in place of *vpr* [36]. If cells are productively infected with this virus and are expressing viral proteins, the HA reporter protein is expressed on the cell surface. Consequently, induction of virus expression from latently-infected cells can be detected by flow cytometry. We have previously found that similar reporter viruses effectively establish latency *in vivo* in humanized mice [10,34,37]. Here, BLT mice were infected for 3–4 weeks then treated with ART (consisting of daily injections of emtricitibine (FTC), tenofovir disoproxil fumarate (TDF), and raltegravir for 2–5 weeks) and suppression of viral loads was monitored by RT-PCR. Mice were then bled to provide a baseline percentage of HA+ human cells in the blood, and subsequently treated with SUW133 by IP injection while maintaining ART (Fig 6A). After an overnight incubation, mice were sacrificed and the percentage of HA+ (HIV-expressing) cells was quantified in the peripheral blood (Fig 6B–6D, and S4 Fig) and spleen (Fig 6E). For spleen, control mice that had been infected and treated with ART but not stimulated with SUW133 were included as unstimulated comparators. In these assays, SUW133 induced significant numbers of latently-infected cells to express viral proteins, as measured by increases in the percentage of HA+ cells after *in vivo* stimulation (Fig 6E).

**Fig 7:**
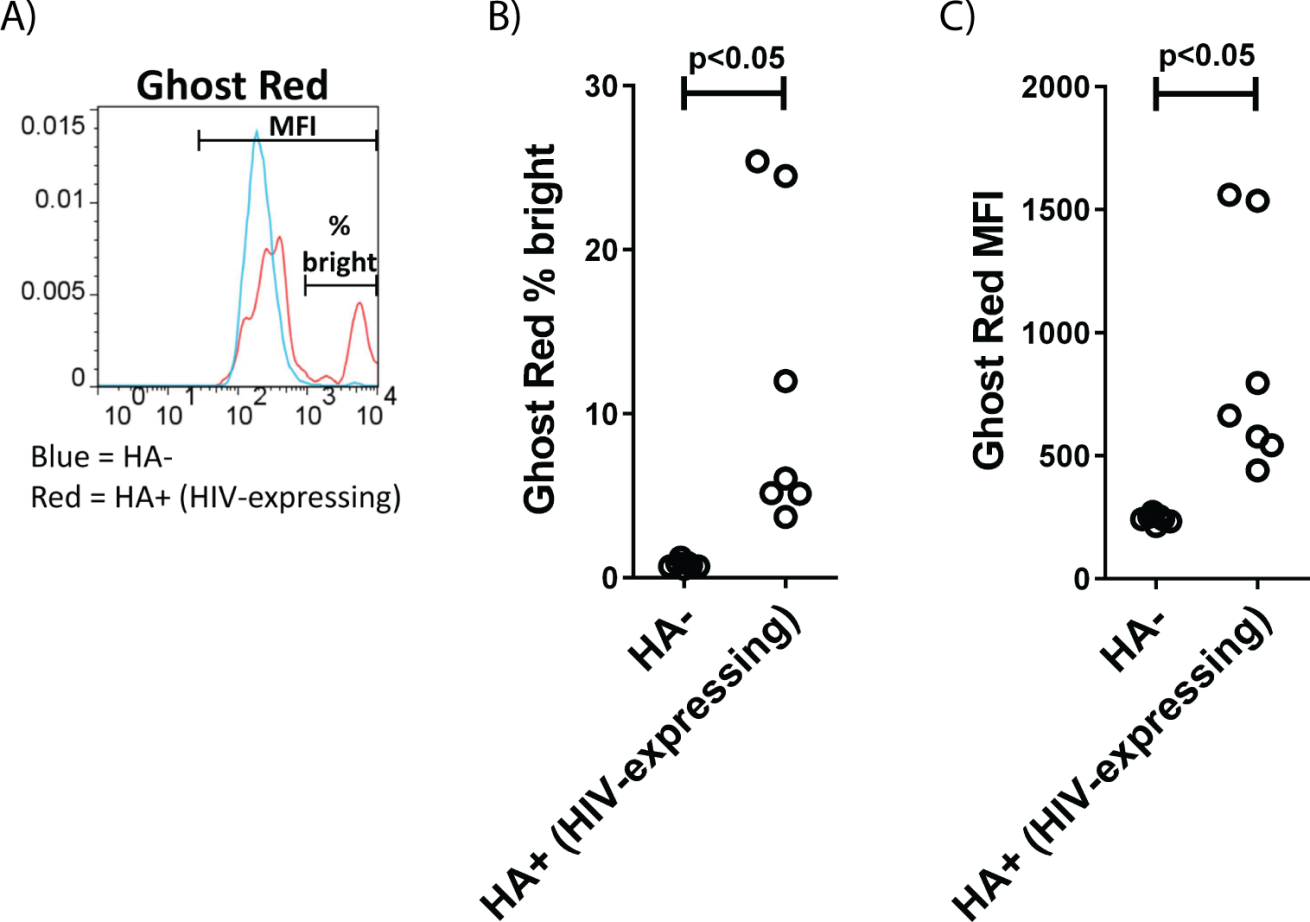
Death of cells reactivated from latency *in vivo*. **A)** Example staining of HA+ (HIV-expressing) versus HA-negative cells using “Ghost Red”, which stains dead or dying cells with disrupted membranes. **B)** Percentage of Ghost Red bright (dead/dying) cells in the HA-negative versus HA+ (HIV-expressing) cells in spleen. **C)** Mean fluorescent intensity of Ghost Red in HA-negative versus HA+ cells in spleen. Each data-point represents an individual mouse. P-values were assessed using a 2-sided Wilcoxon rank sum test for independent data.

An important and as yet unanswered question is whether LRA-induced activation of HIV from latency alone is sufficient to cause death of the host cell, either through viral cytopathic effects or host anti-viral immune responses (anti-HIV immune responses are detectable in BLT mice, [38] although at minimal levels). We therefore included two vital dyes in some of the analysis of infected cells that had been stimulated from latency *in vivo*. Ghost Red and Zombie Yellow dyes are excluded from cells with intact membranes and are enriched in dead or dying cells. We found that within 24 hours of activation, up to 25% of the cells induced to express HIV *in vivo* by SUW133 administration also stained with these cell death dyes, compared with a very low background staining in HA-negative cells from the same animals (Figs 7 and S5), demonstrating that a portion of the cells induced to express HIV proteins *in vivo*, in the absence of an additional “kill” approach, subsequently died.

## Discussion

“Kick and kill” approaches represent promising strategies for eliminating latent HIV and thus contributing to the cure of HIV-infected individuals. Yet, these approaches are limited by a relative lack of safe and effective LRAs that can strongly induce HIV expression *in vivo* [39]. Protein kinase C modulating agents such as bryostatin 1 are among the most effective LRAs to be tested [14]. However, preclinical development of this compound has been hampered by its lack of availability due to its scarce and variable production by its source organism and the attendant logistical, financial, and environmental issues associated with massive harvesting of that source organism from fragile marine reserves. Moreover, due to its complexity and insufficient availability, little has been done to modify the natural product to improve its therapeutic efficacy (with respect to HIV latency reversal) and tolerability (dose-limiting toxicity, myalgia). Indeed, little is known even about whether efficacy and tolerability could be decoupled by such changes in the structure.

Here, we demonstrate that a designed, synthetically accessible PKC activator inspired by bryostatin 1 exhibits a pan-PKC binding selectivity similar to bryostatin 1, better tolerability *in vivo* than the natural product and a more potent capacity to activate HIV from latent reservoirs *ex vivo* and *in vivo*. Significantly, this activation leads to death of some latently-infected cells, thus providing for both “kick” and “kill” activities. It is likely that the observed death of previously latently-infected cells would increase if tested over longer periods of time or with repeated dosing, and could also be further improved by inclusion of therapeutic agents that are specifically intended to kill cells actively expressing HIV, potentially including cell-based therapeutics such as HIV-specific cytotoxic T lymphocytes [11], natural killer cells, or Env-specific antibody based approaches including broadly neutralizing antibodies [8] or immunotoxins [10]. Similarly, the use of LRAs that would synergize with such PKC modulators would be expected to further improve tolerability by dose reduction [31]. Enhancements in delivery such as incorporation of bryologs into CD4-targeted nanoparticles as we have previously described for bryostatin 1 could also represent a more specific and efficient means to reactivate latent virus in CD4+ cells while reducing unwanted side-effects that are mediated through cell types that are not typically HIV-infected [40,41].

Several factors might contribute to the variable level of death observed in those cells actively-expressing HIV (Figs 7 and S5). Data on cell killing were obtained at a single timepoint around 24 hours post-activation. These data thus represent only a snapshot of an ongoing process. It is therefore very likely that additional cells could die at later timepoints. Furthermore, since humanization levels and anti-HIV immune responses are not identical in all animals, variations in the anti-HIV innate and adaptive immune responses in different humanized mice could lead to corresponding variations in the killing of cells that have been recently reactivated from latency. The amount of HIV protein produced in the newly reactivated cells may also in some cases be below the threshold required to trigger viral cytopathic effects that contribute to host cell death. This concept of multifactorial intracellular and extracellular signals contributing to the death of HIV-infected cells during post-integration expression is consistent with the observation that apoptosis in cells harboring pre-integrated virus can be influenced by a variety of different factors including microenvironmental stimuli such as cytokines [42].

Future extensions of the current study would include experiments designed to determine whether this single-dose of LRA is capable of inducing a long-term reduction in the frequency of latently-infected cells in the animals. We also did not directly measure the fraction of HIV proviruses from which SUW133 could reactivate HIV expression, or whether SUW133 is capable of inducing any of the apparently intact proviruses that have proven refractory to reactivation through other means [43]. Future studies could also entail determining the effects of multiple sequential activating events on the latent reservoir. The long-term consequences of inducing even modest levels of T cell activation (as exemplified here by CD69 upregulation without abundant CD25 expression) in essentially all T cells *in vivo* using PKC modulating LRAs are also unknown, and should be considered along with results from acute toxicity experiments in further preclinical studies.

Nevertheless, given the vast body of research directed at naturally occurring LRAs, this proof of principle study, based on one of the most advanced small animal models available for investigating HIV persistence and pathogenesis, establishes that robust induction of HIV expression can be achieved with a designed agent and, significantly, that this agent causes death of some HIV reservoir cells. This study demonstrates how a natural lead can be used as an effective template for the design of a more accessible, efficacious, better tolerated and tunable analog representing now one of the most promising adjuvants for use with ART in strategies to eradicate HIV.

## Materials and methods

### Uninfected human cell isolation and stimulation procedures

Primary human cells from HIV seronegative individuals were cultured in “RF10 medium” consisting of RPMI 1640 medium supplemented with 10% fetal bovine serum (FBS, Omega Scientific), 100 U/mL of penicillin, and 100 μg/mL of streptomycin (Invitrogen). PBMCs were isolated using Ficoll-Paque Plus separation (GE Healthcare). Primary CD4+ T cells were separated from PBMCs by negative immunomagnetic selection using the CD4+ T cell Isolation Kit (Miltenyi Biotec) according to the manufacturer’s instructions. Cells were then exposed to compound for 24 hours before staining and flow cytometric analysis of CD69 levels. Compound incubations were performed by seeding 10^5^ cells/well in 100 μL of RF10 media containing the appropriate concentration of compound in wells of a v-bottomed 96-well plate. Bryostatin 1 (Tocris bioscience) and Prostratin (LC Laboratories) were obtained commercially, and bryologs or prostratin analogs were synthesized as previously described [18,26].

### *In vivo* C57/bl6 mouse experiments

C57/bl6 mice were obtained from the UCLA Department of Radiation Oncology. Compounds were suspended in DMSO and then further diluted in RF10 media to produce a final volume of 500 μL (containing a maximum 4% DMSO). These 500 μL volumes were administered to mice by intraperitoneal injection. Mice were monitored for acute toxicities and then sacrificed at the indicated times. Spleens were removed and disaggregated using a steel mesh, and then filtered through a 40 μm filter to produce a single-cell suspension. The resultant splenocytes were then stained for flow cytometry to analyze surface expression of relevant markers.

### *In vivo* humanized BLT mouse experiments

Humanized bone marrow liver thymus (BLT) mice [32] were constructed by the UCLA humanized mouse core using techniques described previously [34,44,45]. In brief, NOD.Cg-Prkdc^scid^ IL2rg^tm1Wjl^ (Nod-SCID-common gamma chain knockout [NSG]) mice were first irradiated with 270 rads and then transplanted under the kidney capsule with pieces of fetal thymus and liver tissue. Mice were then infused intravenously by retro-orbital injection with 5x10^5^ human fetal liver-derived CD34+ cells isolated by immunomagnetic separation as previously described [46]. At 8 weeks post-transplantation and approximately every 2 weeks thereafter mice were evaluated for reconstitution with human cells. Mice were bled as previously described [34] and peripheral blood mononuclear cells analyzed by flow cytometry. Once reconstituted, mice were infected by retro-orbital injection with HIV strain NL-HA (200ng p24 per mouse), which is a near full-length, pathogenic strain of HIV that encodes a short HA epitope from influenza hemagglutinin in place of *vpr* that is expressed on the surface of productively infected cells [36]. Mice were bled periodically and plasma viral loads monitored by RT-PCR conducted by the UCLA CFAR virology core. Mice were treated with antiretroviral therapy consisting of emtricitibine (FTC), tenofovir disoproxil fumarate (TDF), and raltegravir, essentially as described previously [47], which were administered through daily subcutaneous injection. For mice that were treated with a PKC modulator, the mice were first bled (for pre-stimulation analysis of plasma viral load and HIV expression) and then the PKC modulator was administered by intraperitoneal injection. Because BLT mice are usually less robust than wild-type mice (due to surgery, humanization, and multiple bleeds etc.), the lowest levels of SUW133 in the HIV-infected BLT mice that induced significant activation of biomarker expression in wild-type mice were tested first. The following day (17–24 h after PKC modulator administration) the mice were sacrificed and then PBMC and splenocytes were analyzed by flow cytometry.

### Flow cytometry

During standard flow cytometry staining, samples of 10^5^ cells were suspended in 50 μL of a 1:1 dilution of phosphate buffered saline (PBS):Human AB serum (Sigma). Staining of C57/bl6 mouse splenocytes was performed by adding the following anti-mouse fluorescent conjugated antibodies: CD3-Fluorescein isothiocyanate (FITC, eBioscience 11-0032-82); CD69-Phycoerythrin (PE, eBioscience 12-0691-83); and CD4-Allophycocyanin (APC, eBioscience 17-0041-83). In some experiments CD4-APC was replaced with CD25-APC (eBioscience 17-0251-82) and CD4-Phycoerythrin Cyanin 7 (PC7, eBioscience 25-0041-82). For humanized mouse studies, PBMC and splenocytes from BLT mice were stained with anti-mouse CD45-Alexa 700 (Biolegend 103128) and the following anti-human antibodies: CD45-e450 (eBioscience 48-0459-42); CD3-APC-780 (eBioscience; 47-0038-42); CD4-V500 (BD biosciences; 560768); CD8-Peridinin chlorophyll protein (PerCP)-710 (eBioscience 46-0087-42); CD25-PE (Beckman IM0479U); CD69-APC (Biolegend 310910); CD14-PE-Cy7 (Biolegend 325618). Cells were separately stained for anti-human CD45 e450- (eBioscience 48-0459-42) and anti-HA-Biotin PE (Roche 12158167001) followed by a secondary Streptavidin, R-Phycoerythrin Conjugate (Invitrogen s866) stain. This two-step stain was performed by incubating 1-2x10^6^ cells at 4°C for 20–30 min, washing with 2% FBS in PBS, then adding the secondary stain, incubating again at 4°C for 20–30 min, and washing with 2% FBS in PBS before fixation with 2% paraformaldehyde. In some experiments, cell death was also evaluated by GHOST Red 780 (Tonbo Biosciences 13–0865) or Zombie Yellow (Biolegend 423104) stains. For this procedure, 10^6^ cells were suspended in 100 μL PBS, and then the Zombie yellow and GHOST Red 780 dyes added. Cells were incubated for 20 min at room temperature in the dark and then washed twice with 2% FBS in PBS. Cells were then fixed with 2% paraformaldehyde and incubated for 20 min in the dark. In some experiments cells were then washed with 2% FBS in PBS, resuspended in 1:1 PBS:Human AB serum and then stained for human CD45 and HA, CD3-PE-Cy5 (Biolegend 300310), CD4-ECD (Beckman 6604727), CD8 Per-CO710 (eBioscience 46-0087-42) and CD3-APC (Cell signaling 9602S) as described above.

For human PBMC CD69 induction experiments, cells were stained with CD69-ECD (Beckman Coulter 6607110). During staining, cells were incubated at 4^o^ C for 20 minutes, washed with 2% FBS in PBS, and then resuspended in 2% paraformaldehyde. Stained samples were stored at 4^o^ C.

Flow cytometry samples were analyzed using a Cytomics FC 500 (Beckman Coulter) or a LSR Fortessa (BD Biosciences) flow cytometer. Data were analyzed using FlowJo (v7) software.

### Cytokine quantifications

Quantification of the levels of the murine cytokines IL-2, IL-6, MIP-1-alpha, and TNF-alpha in mouse plasma samples was performed using a custom Milliplex Mouse Cytokine/Chemokine Magnetic Kit (Millipore) according to the manufacturer’s recommendations. Wash steps were performed using a Bio-Plex II Wash Station (Bio-Rad), and the data were acquired using a Bio-Plex 200 System with high-throughput fluidics (Bio-Rad). The resultant data were analyzed using Bio-Plex Manager 6.1 software.

### Statistics

Tests of independent groups were performed using the Wilcoxon Rank Sum test. For hypotheses where we, a priori, expected an increase due to stimulation, we used a 1-sided test. All other tests were two-sided. For mouse and in vitro studies, preliminary results suggested that in most cases a sample size of 3 in each group would be sufficient to observe a significant effect using a 1-sided Wilcoxon rank sum test. Where resources allowed, we added additional mice/replicates to allow for variance in the estimates. BLT mice with fully-suppressed viral loads were included in the analysis. No specific randomization or blinding was performed. For cytokine analysis, where cytokine concentration values were below the limit of detection, a value that was midway between 0 and the limit of detection was used. Paired data was analyzed using the Wilcoxon Signed Rank Test for paired data. If, a priori, we expected a decrease, we used a 1-sided test, otherwise we used a two-sided test. The figure legends state specifically if a test is 1 or 2 sided. Linear regression was used to assess the relationship between CD69 expression and latency activation. Residual plots and the R^2^ value suggest a good fit to the model.

### U1 cell stimulations

U1 cells [48] (tested negative for bacteria, mold, yeast, and mycoplasma) were obtained through the AIDS Reagent Program, Division of AIDS, NIAID, NIH from Dr. Thomas Folks. Cells and compound were seeded in a final 200 μL volume of RF10 media at a cell density of 20,000 cells/well in v-bottomed 96 well plates and incubated at 37°C for 48 hours. After incubation, cells were pelleted and supernatant harvested then diluted in a 2% Triton-x-100/phosphate buffered saline solution. The concentration of HIV p24 protein in the culture supernatant was quantified using the HIV p24 antigen enzyme linked immunosorbent assay (ELISA) kit (Beckman Coulter) according to the manufacturer’s instructions.

### J-Lat cell stimulations

J-Lat cells (clone 10.6) [49] (tested negative for bacteria, mycoplasma, and fungi) were obtained through the AIDS Reagent Program, Division of AIDS, NIAID, NIH from Dr. Eric Verdin. Cells were incubated in 100 μL volume of RF10 medium containing the indicated concentrations of compound for 48h before analysis. Stimulations were performed in v-bottomed 96-well tissue culture plates with a starting cell density of 25000 cells/well. During harvesting, cells were washed and resuspended in 2% paraformaldehyde. The percentage of cells expressing GFP was then quantified by flow cytometry using a FC 500 flow cytometer (Beckman Coulter) and FlowJo software (version 7.6).

### Clinical specimens

Nine HIV-infected individuals receiving ART for a median of 2.6 years (range 0.4–9.1) were studied. All study subjects maintained undetectable levels of plasma viremia (<50 copies/mL) at the time of study (S1 Table).

### Preparation and incubation of resting CD4^+^ T cells with study compounds

CD4^+^ T cells were isolated from cryopreserved peripheral blood mononuclear cells (PBMCs) using a cell enrichment kit (StemCell Technologies). For patient samples 1–6, resting CD4^+^ T cells were further isolated by depleting CD25^+^, HLA-DR^+^ and CD69^+^ CD4^+^ cells using PE-conjugated antibodies (BD Biosciences) and anti-PE microbeads (Miltenyi Biotec). CD4^+^ T cells were incubated with RPMI 1640-based medium containing antiretroviral drugs consisting of T-20 (100 μg/mL), tenofovir (1 μM), and emtricitabine (1 μM), and test stimuli at concentrations indicated in the figures.

### Quantitation of HIV RNA in ex vivo patient samples

The copy number of virion-associated HIV RNA in the cell culture supernatants was measured using the Cobas Ampliprep/Cobas Taqman HIV-1 Test, Version 2.0 (Roche Diagnostics) following 36–48 hours of incubation of cells with the above study compounds. The limit of detection was 20 copies/mL.

### Source of PKC analog

SUW133 corresponds to analog 4 in a previous study describing its synthesis [26].

The final *Ki* data for each of the isoforms for SUW133 is as follows:

alpha: 1.8 nM
beta I: 6.3 nM
gamma: 5.6 nM
delta: 2.0 nM
epsilon: 5.8 nM
eta: 6.4 nM
theta: 2.0 nM

#### PKC binding assay protocol

The protein kinase C affinity of test compounds was performed via competition with ^3^H-phorbol-12,13-dibutyrate (^3^H-PDBu) as described below. This entails a glass-fiber filtration method to determine bound radioligand.

#### Preparation of the binding assay buffer

To a polypropylene vial was added Tris-HCl (pH 7.4, 1 M, 1 mL), KCl (1 M, 2 mL), CaCl_2_ (0.1 M, 30 μL), and bovine serum albumin (40 mg, from Sigma). This mixture was diluted to 20 mL with deionized H_2_O, and mixed gently. The buffer was stored on ice until use. The final concentration of these constituents are shown in S2 Table.

#### Preparation of phosphatidylserine (PS) vesicles and PKC solution

For every 2 assays, 3.5 mg phosphatidylserine (Avanti Polar Lipids, porcine, obtained as a solution in CHCl_3_) was isolated by removing chloroform under a stream of nitrogen followed by reduced pressure. The solid PS was suspended as vesicles in binding assay buffer (3.5 mL) by sonicating (Branson Sonifier 250, power = 2, 50% duty cycle) six times for 30 s with a 30 s rest between sonications. The resulting cloudy mixture (1 mg/mL PS) was stored on ice until use.

Assay Protein Kinase C (PKC) was prepared by dissolving a 4 μg aliquot of the indicated recombinant human PKC isoform (Invitrogen) into 11.6 mL of binding assay buffer. The *K*_*D*_ values for all 7 isoforms were determined experimentally. The diluted PKC was stored on ice for immediate use. This amount is sufficient for 2 assays with room for error.

#### Preparation of compound and ^3^H-PDBu dilutions

^3^H-PDBu (American Radiolabeled Chemicals, Inc.; specific activity: 20 μCi/mmol) was diluted 10-fold in DMSO from a 1 mCi/mL commercial acetone solution. This 500 nM stock solution was further diluted in DMSO to 30 nM for use in assays. The 500 nM and 30 nM stock solutions were stored frozen in DMSO at −20°C until use. Compound dilutions were also prepared in DMSO, serially diluting from a chosen high concentration (depending on analog potency) by factors of 3 to 8. Seven analog concentrations were used to define the inhibition curve. For SUW133, for instance, concentrations of sample dilutions were 667 nM, 222 nM, 74.0 nM, 24.7 nM, 8.23 nM, 2.74 nM, and 0.914 nM.

#### Master mix solution

To a polypropylene tube 3.3 mL of 1 mg/mL phosphatidylserine vesicles solution, 11 mL of PKC isoform solution, and 1.1 mL of 30 nM ^3^H-PDBu solution were added. The resulting solution was vortexed to mix and stored on ice.

#### Assay protocol

Triplicate data points were obtained for each analog concentration. For each data point, 280 μL of Master Mix Solution and test compound at the given concentration (20 μL) were added to a polypropylene tube. Non-specific ^3^H-PDBu binding was assessed in triplicate by substitution of test compound with unlabeled PDBu (20 μL of a 75 μM stock, assay concentration: 5 μM). Maximal ^3^H-PDBu binding was analyzed in triplicate by substitution of test compound with 20 μL DMSO. The solutions were vortexed to mix, incubated at 37°C for 10 min, and incubated on ice for at least 30 min prior to filtration. Glass-fiber filters (Whatman GF/B) were prepared by soaking in a solution of aqueous polyethyleneimine (10% by volume, 18 mL) diluted in water (600 mL) for ≥60 min. Rinse buffer (500 mL, 20 mM Tris, pH 7.4) was cooled to 4°C for the duration of the incubation period and for the remainder of the assay. Assay tube contents were vacuum-filtered through the polyethylenimine-soaked filters, using a Brandel Harvester, washing with rinse buffer three times and allowing to dry first under vacuum filtration for 5 minutes and then under ambient conditions for ≥ 2 hours. The resulting dry filters had circular perforations for each data point which were removed with forceps and placed into a scintillation vial. Scintillation vials were filled with Bio-Safe II scintillation fluid (5 mL) and were measured for radioactivity using a Beckman LS 6000SC scintillation counter. Counts per minute (cpm) were averaged for each triplicate dilution. The data were then plotted (cpm vs. log(concentration)) using Prism by GraphPad Software and an IC_50_ was determined using that program’s built-in one-site competition least squares regression function. K_i_ values were calculated by the equation:

$$K_{i}=IC_{50}/\left(1+\left(\left[{}^{3}H-PDBu\right]/K_{d}\right)\right)$$

The K_d_ of ^3^H-PDBu was determined via saturation binding under identical conditions and the values for each PKC isoform are as follows: **α = 15.1 nM; βI = 8.8 nM; γ = 13.8 nM; δ = 4.5 nM; ε = 6.2 nM; η = 18.4 nM; θ = 28.8 nM.**

### Ethics statement

#### Human subject research

For experiments involving the ex vivo stimulation of latent HIV from patient-derived cells: Leukapheresis were conducted in accordance with clinical protocols approved by the Institutional Review Boards of the National Institute of Allergy and Infectious Diseases at the National Institutes of Health. All study subjects provided written informed consent. Additional human cells/tissues used in this study were from anonymous sources and therefore are not considered human subjects and thus not subject to Institutional Review Board review. Anonymized fetal tissue was obtained from either the UCLA Gene and Cellular Therapy Core or Advanced Biosciences Resources Inc. Peripheral blood was obtained through the UCLA Blood Bank in an anonymous fashion.

#### Animal research

Animal experiments conformed to all local and national guidelines and regulations (including the Public Health Service Policy on Humane Care and Use of Laboratory Animals, the Guide for the Care and Use of Laboratory Animals, and the AVMA Guidelines for the Euthanasia of Animals), and procedures were approved by the UCLA Animal Research Committee (Approval number ARC # 1996–058). Euthanasia was performed by anesthetizing animals with isofluorane followed by cervical dislocation.

## Supporting information

S1 Fig Activation of HIV from latency in the U1 cell line, as measured by the presence of HIV p24 protein in the supernatant following a 48h incubation period. Data from 6–11 individual replicates derived from 5 independent experiments (Error Bars = SEM).
(TIF)

S2 Fig Percentage of murine CD3+ T cells in spleen that also express CD25 at different times after administration of 10 μg/mouse of SUW133 to C57/bl6 mice (each data-point represents a different animal). (TIF)

S3 Fig Interleukin 6 (IL-6) levels in the plasma of mice described in Fig 4 parts A and B. IL-6 levels were elevated for SUW133, but declined significantly between days 1 and 9 post-injection (p = 0.05, 1-sided Wilcoxon signed rank test for paired data). Error Bars = SEM, N = 4 mice/group for control and 3 mice/group for SUW133.
(TIF)

S4 Fig Example HA expression data from the blood of 3 animals before and after (following overnight incubation) *in vivo* administration of 2 μg SUW133. (TIF)

S5 Fig Death of HIV-expressing cells as quantified using Zombie Yellow vital dye. Each data-point represents a different mouse (same animals as shown in Fig 7). P-values were assessed using a 2-sided Wilcoxon rank sum test for independent data.
(TIF)

S1 Table Study Subject Information. (DOC)

S2 Table PKC binding assay buffer composition. (DOC)
