# Supplementary material for: In vivo activation of latent HIV with a synthetic bryostatin analog effects both latent cell "kick" and "kill" in strategy for virus eradication
Source: PLoS Pathog. 2017 Sep 21;13(9):e1006575. doi: 10.1371/journal.ppat.1006575 (PMC5608406; doi:10.1371/journal.ppat.1006575)
Supplement: S1 Table — (DOC) [file ppat.1006575.s006.doc]

**Table S1. Study Subject Information.**

| **Subject** | **Duration on ART (years)** | **CD4 (%)** | **CD4 count (/μl)** | **CD8 (%)** | **CD8 count (/μl)** | **Plasma viremia (RNA copies/ml)** |
| --- | --- | --- | --- | --- | --- | --- |
| **1** | 4.5 | 41 | 771 | 42 | 790 | <50 |
| **2** | 2.0 | 53 | 1468 | 36 | 997 | <50 |
| **3** | 6.3 | 38 | 935 | 48 | 1181 | <50 |
| **4** | 2.5 | 44 | 577 | 43 | 563 | <50 |
| **5** | 2.6 | 30 | 342 | 37 | 421 | <50 |
| **6** | 0.9 | 30 | 564 | 43 | 808 | <50 |
| **7** | 9.1 | 37 | 760 | 47 | 965 | <40 |
| **8** | 0.4 | 31 | 549 | 35 | 619 | <40 |
| **9** | 4.4 | 40 | 809 | 42 | 849 | <40 |

Duration of antiretroviral therapy (ART), percentage of PBMC expressing CD4 or CD8 surface markers, CD4 and CD8 counts, and viral loads are provided for the nine subjects.
