# Supplementary material for: In vivo activation of latent HIV with a synthetic bryostatin analog effects both latent cell "kick" and "kill" in strategy for virus eradication
Source: PLoS Pathog. 2017 Sep 21;13(9):e1006575. doi: 10.1371/journal.ppat.1006575 (PMC5608406; doi:10.1371/journal.ppat.1006575)
Supplement: S2 Table — (DOC) [file ppat.1006575.s007.doc]

Table S2. PKC binding assay buffer composition

| **Constituent** | **Stock concentration** | **Quantity** | **Final Concentration** |
| --- | --- | --- | --- |
| **pH 7.4 Tris-HCl** | 1.0 M | 1.0 mL | 50 mM |
| **KCl** | 1.0 M | 2.0 mL | 100 mM |
| **CaCl_2_** | 0.10 M | 30 μL | 0.15 mM |
| **Bovine Serum Albumin** | - | 40 mg | 2 mg/mL |
| **Deionized H_2_O** | - | Final vol of 20 mL | - |

(Note quantities can be doubled for larger batches)
